# Editorial: Photobiomodulation therapy for brain disorders, volume II

**DOI:** 10.3389/fneur.2026.2009205

**Published:** 2026-10-12

**Authors:** Qianting Deng, Ge Song, Luodan Yang

**Affiliations:** Laboratory of Exercise and Neurobiology, School of Physical Education and Sports Science, South China Normal University, Guangzhou, China

**Keywords:** autism spectrum disorder, brain disorders, neuroinflammation, photobiomodulation, stroke, traumatic brain injury

Photobiomodulation (PBM, formerly also known as low-level laser therapy) represents a rapidly evolving non-pharmacological neuromodulation strategy for a broad spectrum of brain pathologies, ranging from chronic neurodegenerative diseases to acute brain injury, post-stroke sequelae, and neurodevelopmental conditions (1–3). This Research Topic compiles four accepted articles that collectively expand our understanding of PBM, spanning a mechanistic narrative review, sport-related concussion clinical pilot trial, scoping review of post-stroke dysfunction clinical evidence, and a hypothesis-generating pediatric case report in autism spectrum disorder (ASD). The Research Topic illuminates shared molecular pathways and translational bottlenecks, encouraging preliminary clinical signals as well as critical unresolved questions for future mechanistic and clinical investigation.

Blivet et al. deliver a comprehensive mini-review entitled “*Photobiomodulation as a Regulator of Neuroinflammation: Mechanistic Insights and Therapeutic Potential in Brain Disorders*,” framing neuroinflammation as a unifying pathological hallmark across heterogeneous brain disorders including Alzheimer's disease (AD), Parkinson's disease (PD), multiple sclerosis (MS), major depressive disorder (MDD), traumatic brain injury (TBI) and stroke. Pre-clinical data consistently demonstrate multi-target anti-inflammatory effects of PBM, including shifting microglia from pro-inflammatory toward regulatory phenotypes, suppressing NLRP3 inflammasome-driven cytokine amplification, reinforcing Nrf2-dependent antioxidant defense, preserving blood-brain barrier integrity, and up-regulating neurotrophic factors such as brain-derived neurotrophic factor (BDNF) (3, 4). Notably, this work draws attention to the brain-gut axis as an alternative therapeutic route, demonstrating that combined brain-gut PBM can produce robust neuroprotective outcomes independent of sufficient transcranial light penetration into deep cerebral parenchyma. Importantly, the review highlights substantial divergence between pre-clinical promise and clinical observations. Encouraging signals exist for AD and MS, whereas primary outcome results remain largely negative in PD and major depressive disorder. Major translational obstacles are emphasized, including heterogeneous irradiation parameters across studies, insufficient standardization of dosimetry, absence of validated target-engagement biomarkers, and limited light penetration through human scalp and skull. The authors propose that PBM may be best positioned as adjunctive rather than stand-alone therapy for most brain-related indications, calling for well-powered, sham-controlled clinical trials to validate efficacy.

Moving from mechanistic synthesis toward real-world acute injury settings, Chermann et al. report the RECOVERY Study, a randomized single-blind pilot clinical trial evaluating a combined brain-PBM plus static magnetic field device (RGn550) for adults suffering acute sport-related concussion within 72 h post-injury. Fifty athletes were randomized to receive 5 Hz or 10 Hz pulsed-light stimulation for two 20-min treatment sessions (Day 0 and Day 7), with follow-up extending to Day 52. The primary safety endpoint showed that both 5 Hz and 10 Hz stimulation modalities exhibited acceptable tolerability. All adverse device-related events were mild and spontaneously resolved, predominantly somnolence and local device-site discomfort, without serious adverse events. No statistically significant differences were detected between the two frequency groups for safety or exploratory secondary endpoints. Interesting within-visit improvements were observed in oculomotor metrics such as near-point of convergence (NPC), alongside changes in balance, executive function, concussion-related symptom scores, and glial fibrillary acidic protein (GFAP) biomarker levels. Given the lack of a sham-control arm, these functional and biomarker shifts cannot be causally attributed to intervention and remain hypothesis-generating. Nevertheless, this pilot establishes critical feasibility data, effect-size estimates, and methodological reference for designing definitive double-blind sham-controlled trials for acute sport-related concussion, a clinical area currently lacking disease-modifying interventions.

Ren et al. systematically summarize contemporary clinical evidence of PBM for diverse post-stroke functional impairments. Two major application paradigms are distinguished as transcranial PBM (tPBM) and peripheral PBM over paretic limbs. Transcranial PBM has yielded promising preliminary results for post-stroke cognitive impairment and aphasia, while peripheral direct irradiation of paretic limbs ameliorates limb spasticity, muscle fatigue, and hemiplegic shoulder pain. Mechanistically, transcranial PBM improves cerebral blood flow, supports neurogenesis and synaptogenesis, while peripheral PBM optimizes muscle metabolism, enhances muscle-fiber recruitment, and exerts analgesic effects. Still, several translational barriers persist: large variability in wavelength, power density, irradiation site, and treatment schedules among clinical trials; substantial inter-species differences in skull light-penetration capacity that complicate extrapolating rodent pre-clinical outcomes to human patients. The authors point out that peripheral PBM might be more clinically implementable as an adjuvant rehabilitation intervention, and propose combinatorial strategies merging PBM with other non-invasive brain stimulation or robot-assisted rehabilitation for post-stroke recovery.

Adding to this Research Topic, Zaharakis and Bogner present a brief case report which expands PBM research into neurodevelopmental disorder territory. This single-subject observational work describes temporal association between pulsed transcranial 660 nm PBM intervention and marked elevation of urinary aluminum excretion in a 5-year-old boy diagnosed with ASD. Urinary aluminum increased approximately 10-fold relative to stable baseline levels during intervention and stayed above reference range at 2-month follow-up. The authors explicitly stress key interpretative caveats. This temporal correlation cannot prove a causal link, and elevated urinary aluminum cannot confirm tissue-metal mobilization, reduced systemic burden, or clinical therapeutic benefit. Multiple confounding factors, including hydration status, renal tubular handling, urine pH, and environmental exposure, may affect urinary metal readouts. This report generates new mechanistic hypotheses about potential PBM modulation of metal-excretion pathways via mitochondrial-dependent ATP-driven transporters and glutathione homeostasis, while strongly advocating for rigorously controlled trials with independent dosimetry verification, systematic dietary-exposure documentation and multi-modal biomarkers to further test these preliminary observations.

## Outlook and concluding remarks

Collectively, the four manuscripts within this Research Topic advance our understanding of PBM from molecular neuroinflammatory mechanisms across multiple brain diseases, through acute-trauma and post-stroke clinical pilot experiences, to exploratory observations in pediatric neurodevelopmental disorder. Consistent themes emerge across all contributions: (1) Pre-clinical findings demonstrate robust multi-target neuroprotective, anti-inflammatory effects of PBM, yet clinical translation faces major hurdles of dosimetric heterogeneity and limited transcranial light penetration in human beings; (2) Brain-gut peripheral delivery strategy represents a promising alternative route bypassing skull-penetration limitations and deserves further pre-clinical and clinical validation; (3) Safety profiles across different populations (adult athletes, stroke survivors, pediatric ASD subject) appear favorable, but efficacy evidence remains preliminary for most indications, reinforcing the necessity for well-designed sham-controlled trials; (4) There is an urgent unmet need for validated target-engagement biomarkers to bridge pre-clinical mechanistic discoveries and clinical outcome measurements. We anticipate that findings assembled in this Research Topic will inspire further optimized mechanistic research, standardized PBM protocol development, and high-quality clinical investigations to clarify the real therapeutic potential of photobiomodulation as non-invasive adjuvant therapy for diverse brain disorders.
